# Suppression of neutrophils by sodium exacerbates oxidative stress and arthritis

**DOI:** 10.3389/fimmu.2023.1174537

**Published:** 2023-08-02

**Authors:** Leticija Zlatar, Aparna Mahajan, Marco Muñoz-Becerra, Daniela Weidner, Galyna Bila, Rostyslav Bilyy, Jens Titze, Markus H. Hoffmann, Georg Schett, Martin Herrmann, Ulrike Steffen, Luis E. Muñoz, Jasmin Knopf

**Affiliations:** ^1^ Department of Internal Medicine 3 – Rheumatology and Immunology, Friedrich-Alexander-University Erlangen-Nürnberg (FAU) and Universitätsklinikum Erlangen, Erlangen, Germany; ^2^ Deutsches Zentrum für Immuntherapie (DZI), Friedrich-Alexander-University Erlangen-Nürnberg and Universitätsklinikum Erlangen, Erlangen, Germany; ^3^ Department of Histology, Cytology, Embryology, Danylo Halytsky Lviv National Medical University, Lviv, Ukraine; ^4^ Division of Nephrology and Hypertension, Universitätsklinikum Erlangen, Erlangen, Germany; ^5^ Programme in Cardiovascular and Metabolic Disorders, Duke-NUS Medical School, Singapore, Singapore; ^6^ Department of Dermatology, Allergology, and Venereology, University of Lübeck, Lübeck, Germany; ^7^ Department of Pediatric Surgery, University Medical Center Mannheim, University of Heidelberg, Mannheim, Germany

**Keywords:** neutrophils, sodium chloride, reactive oxygen species, neutrophil extracellular traps (NETs), osteoclasts, K/BxN serum transfer arthritis

## Abstract

**Introduction:**

Typical Western diet, rich in salt, contributes to autoimmune disease development. However, conflicting reports exist about the effect of salt on neutrophil effector functions, also in the context of arthritis.

**Methods:**

We investigated the effect of sodium chloride (NaCl) on neutrophil viability and functions *in vitro*, and *in vivo* employing the murine K/BxN-serum transfer arthritis (STA) model.

**Results and discussion:**

The effects of NaCl and external reactive oxygen species (H_2_O_2_) were further examined on osteoclasts *in vitro.* Hypertonic sodium-rich media caused primary/secondary cell necrosis, altered the nuclear morphology, inhibited phagocytosis, degranulation, myeloperoxidase (MPO) peroxidation activity and neutrophil extracellular trap (NET) formation, while increasing total ROS production, mitochondrial ROS production, and neutrophil elastase (NE) activity. High salt diet (HSD) aggravated arthritis by increasing inflammation, bone erosion, and osteoclast differentiation, accompanied by increased NE expression and activity. Osteoclast differentiation was decreased with 25 mM NaCl or 100 nM H_2_O_2_ addition to isotonic media. In contrast to NaCl, external H_2_O_2_ had pro-resorptive effects *in vitro*. We postulate that in arthritis under HSD, increased bone erosion can be attributed to an enhanced oxidative milieu maintained by infiltrating neutrophils, rather than a direct effect of NaCl.

## 1 Introduction

Neutrophils are the most abundant white blood cells, and the first line of defense in innate immunity. Upon activation, they migrate to the site of inflammation, to execute various effector functions: phagocytosis, degranulation, reactive oxygen species (ROS) and cytokine production, and neutrophil extracellular traps (NETs) release (1, 2). In neutrophils, azurophilic (primary) granules containing enzymes such as myeloperoxidase (MPO) or neutrophil elastase (NE) fuse with the phagosome during phagocytosis. MPO is a heme enzyme with peroxidase-like and halogenizing activities (3), it utilizes the H_2_O_2_ as a substrate to generate hypochlorous acid (HClO). The latter kills pathogens on the expense of tissue destruction (4). The formation of NETs is commonly initiated in neutrophils undergoing an oxidative burst (5), followed by chromatin de-condensation and nuclear membrane disintegration (6), NE and MPO access to the nucleus, histone modification, cellular membrane rupture, and chromatin expulsion (7). NETs can exert pro- or anti-inflammatory activities depending on the intensity of neutrophil infiltration, and are eventually dismantled by serum DNases and cleared by phagocytes.

Neutrophils contribute to the pathogenesis of many diseases. One example is rheumatoid arthritis (RA) (8), a chronic inflammatory autoimmune disease characterized by persistent and progressive joint and synovial membrane damage (2, 7). In patients with RA, immune complexes deposited in synovial tissues trigger inflammatory responses driven by neutrophils and NETs (2). A well-established murine model for RA is K/BxN serum transfer arthritis (STA) (7, 8). The inflammation after transfer of serum or purified glucose-6-phosphate isomerase (G6PI)-specific IgGs from arthritic transgenic K/BxN mice is driven by autoantibodies against G6PI (9). In this model neutrophils are an essential player in the pathogenesis (10).

A Western diet, rich in salt, has various effects on the innate immune system. It has long been considered a risk factor for autoimmune diseases (11–14). NaCl aggravates inflammatory arthritis via induction of pathogenic CD4+ T helper cells which produce interleukin-17 (IL-17) (15). The effect of salt on neutrophils, however, hasn´t been fully elucidated, as many publications provide conflicting information. It was reported that NaCl-induced hyperosmolarity suppresses some leukocyte functions, such as chemotaxis, phagocytosis, intracellular killing of bacteria and superoxide production (16). More recently, it was reported that a high salt environment suppresses ROS-dependent NET formation (17) while Krampert et al. reported that “high salt” (40 mM additional NaCl) reduced neutrophil movement, degranulation and ROS production with no changes in cell viability or NET formation. The latest publication showed that the effect of additional 50 mM NaCl was time-dependent and caused a delayed activation of human neutrophils. In short-term and long-term neutrophil cultures, production of IL-8 and respiratory burst were inhibited or augmented, respectively (18).

The ionic strength in the bodies of mammalians is strictly regulated (19). The overall ionic strength of cell bodies and interstitial fluids is approximately 150 mM corresponding to 300 mOsm/kg. Under physiological conditions, sodium plays an important role in maintaining the volume of extracellular fluids and generating cells’ membrane potential (20). It also plays an important role in muscle contraction and is directly related to blood pressure (21). In the kidney, which is involved in osmoregulation (22), this osmotic tonicity is locally greatly exceeded. On the way of the urine from the blood to the inner medulla osmolarity gradually increases from 300 to 1200 mOsm/kg. The tonicity of the corresponding interstitial spaces follows the increase inside the tubules (23). In addition to these osmolar active ions, bones constitutively contain much sodium adsorbed to the apatite in an osmolar inactive manner. *In vivo* sodium quantification in the healthy human wrist by the means of MRI revealed average sodium concentrations ranging from 115 to 150 mmol/L in noncartilaginous regions, and from 200 to 210 mmol/L in cartilaginous regions (24). However, the exact tissue concentration of sodium in many pathological tissues, as in those of RA patients, has not been determined yet. In patients with human brain tumors, MRI showed increased sodium concentration in tumors relative to that in normal brain structures (25). High sodium intake has been associated with rheumatoid arthritis (26), and patients with rheumatoid arthritis showed increased sodium excretion (27). Furthermore, hypertonic sodium rich environment can be found in the lymphatic organs, skin, and the inflamed tissue, under both physiological and pathological conditions. In inflamed tissues of patients with skin infection the sodium accumulates at the site of infection (23, 28). Whether this sodium increases the local tonicity is not fully clarified. Measurement of the tissue osmolality in lymphoid tissues also revealed the hyperosmolar environment (29).

Here we examined the effects of hypertonic sodium-rich media on neutrophil viability, nuclear morphology and various cell activities, as well as osteoclast differentiation and resorption activity, in the context of inflammatory arthritis. We observed that hypertonic sodium-rich media changed the morphology of neutrophils’ nuclei, greatly reduced neutrophil viability by primary or secondary necrosis, and inhibited neutrophil effector functions, except, it fostered ROS production. *In vivo*, HSD aggravated STA by increasing tissue inflammation, bone erosion and osteoclast count, and was accompanied by enhanced neutrophil infiltration in the hind paws. Additional 25 mM NaCl decreased osteoclast differentiation *in vitro* without altering their resorption activity, whereas external ROS (H_2_O_2_) increased resorption activity, despite decreased cell differentiation in these conditions.

## 2 Materials and methods

### 2.1 Ethical issues

Investigations on human material were performed in accordance with the Declaration of Helsinki and with the approval of the ethical committee of the University Hospital Erlangen (permit 243_15 B). A written informed consent was given by each donor. All animal experiments and procedures were performed according to institutional guidelines on animal welfare and were approved by the local Animal Care and Use Committees of the Danylo Halytsky Lviv National Medical University (permit numbers 20191219/10 and 20201221/9).

### 2.2 Isolation of human neutrophils

Whole blood from healthy human donors was freshly drawn into EDTA tubes (Sarstedt). We isolated neutrophils by density gradient centrifugation at 350 g for 30 minutes at room temperature (RT) with Lymphoflot Ficoll-Diatrizoate (Bio-Rad). The high-density layer was subjected to hypotonic erythrocyte lysis. We resuspended pelleted neutrophils in Dulbecco´s Phosphate Buffered Saline (DPBS, ThermoFisher Scientific) and determined the viable cell concentration by staining with acridine orange/propidium iodide (Logos Biosystems) employing the Luna-FL™ Dual Fluorescence Cell Counter (Logos Biosystems).

### 2.3 4 color death staining

We incubated freshly isolated neutrophils in DPBS-NaCl buffers at various osmolarities (1) isotonicity 137 mM NaCl, (2) moderate hypertonicity 200 mM NaCl, and (3) high hypertonicity 300 mM NaCl, with or without addition of 10% heat-inactivated fetal calf serum (FCS, c.c.pro GmbH), at different time points. Corresponding buffers were prepared by addition of 3.4 M NaCl solution (Merck) to the standard DPBS buffer. Cell viability was assessed by Gallios Flow Cytometer (Beckman Coulter) after 30-minute staining with Annexin A5-FITC (Immunotools, 0.5 µg/ml), PI (Sigma-Aldrich, 1 µg/ml), DilC1(5) (Invitrogen, 1.67 nM) and Hoechst33342 (Molecular Probes, 1 µg/ml) in Ringer´s solution (Delta Select) at 4°C as reported previously (30). The analysis was performed in Kaluza Analysis 2.1 software (Beckman Coulter).

### 2.4 ROS and mitochondrial ROS production

We pre-incubated freshly isolated neutrophils at 37°C with general oxidative stress indicator 2 µM CM-H2DCFDA (ThermoFisher Scientific) or mitochondrial superoxide indicator 5 µM MitoSOX Red (ThermoFisher Scientific) for 20 or 10 minutes, respectively, and incubated them in respective DPBS-NaCl buffers (137, 200 or 300 mM) without serum upon Phorbol 12-myristate 13-acetate (PMA, 100 ng/mL) or Pyocyanin (10 µM or 50 µM, respectively) stimulation for another 20 minutes. We stained the neutrophils with CD15 APC (BioLegend, 1:150) and CD16 PB (BD Pharmingen, 1:150) for 30 minutes at 4°C, and assessed the CM-H2DCFDA (ThermoFisher Scientific) and MitoSOX Red (ThermoFisher Scientific) fluorescence in the FL-2 channel with the Gallios Flow Cytometer (Beckman Coulter). The analysis was performed in Kaluza Analysis 2.1 software (Beckman Coulter).

### 2.5 Phagocytosis

We coated the Fluoresbrite YG Carboxylate Microspheres, 1 µm (Polysciences, 15702-10) with 2 mg/mL human immunoglobulins (IVIg, Gammunex-C) or 2 mg/mL human serum albumin (HSA, Sigma-Aldrich) and stored them at 4°C in a DPBS-NaCl buffer with 10 mg/mL bovine serum albumin (BSA, Santa Cruz Biotechnology). We then centrifuged the microspheres and replaced the supernatant with 50% autologous serum diluted in DPBS-NaCl buffers of various salt concentration (137, 200 or 300 mM). We vortexed the microspheres for 5 minutes and washed them with 1% BSA in DPBS. We resuspended them in hypertonic sodium-rich media (137, 200 or 300 mM). The microspheres were sonicated for 10 minutes, and vortexed for 5 minutes before immediate use. We isolated the neutrophils as described in 2.2., and resuspended them in respective DPBS-NaCl buffers (137, 200 or 300 mM). We added the final microsphere suspension to the cells and incubated them for 1 hour at 37°C and 5% CO_2_. We stained the cells with CD15 APC (BioLegend, 1:150) and CD16 PB (BD Pharmingen, 1:150) for 30 minutes at 4°C and quantified the signal using the Gallios Flow Cytometer (Beckman Coulter). The analysis was performed in Kaluza Analysis 2.1 software (Beckman Coulter).

### 2.6 Degranulation

We isolated neutrophils as described in 2.2., and incubated them for 1 hour with PMA (100 ng/mL) or Pyocyanin (10 µM) in various DPBS-NaCl buffers (137, 200 or 300 mM). We stored the supernatant and later used it for MPO or NE activity measurement (2.7 and 2.8, respectively). The remaining cell pellet was resuspended and stained with anti-CD66b-FITC (Immunotech, 1:1000) for 30 minutes at 4°C and quantified using the Gallios Flow Cytometer (Cytoflex S) to assess degranulation. The analysis was performed in Kaluza Analysis 2.1 software (Beckman Coulter).

### 2.7 MPO activity

To assess the MPO peroxidation activity, reaction was developed by adding the TMB substrate set (BioLegend) to the collected supernatants. The reaction was stopped by adding 25% sulfuric acid (PanReac AppliChem). The optical densities (ODs) were read at 450 nm with a reference at 620 nm using the SUNRISE microplate reader (Tecan). To assess the MPO chlorination activity, we used the EnzChek™ Myeloperoxidase (MPO) Activity Assay Kit (ThermoFisher Scientific) according to manufacturer´s instructions. The fluorescence intensity was read in an Infinite F200 PRO plate reader (Tecan; ex. 485 nm, em. 530 nm). We used RIPA buffer (ThermoFisher Scientific) for complete cell lysis representing maximum MPO activity in the cell supernatant.

### 2.8 NE activity

To assess the NE activity, reaction was developed by adding the NE fluorogenic substrate (MeOSuc-AAPV-AMC, Santa Cruz Biotechnology, 1:10) to the collected supernatants. The fluorescence intensity was measured for 12 hours at 37°C in an Infinite F200 PRO plate reader (Tecan; ex. 360 nm, em. 465 nm). We used RIPA buffer (ThermoFisher Scientific) for complete cell lysis representing maximum NE activity in the cell supernatant. Endpoint values were analyzed.

### 2.9 Quantification and imaging of NETs

We employed SYTOX™ Green Nucleic Acid stain (ThermoFisher Scientific, 1: 1200) to detect extracellular DNA (ecDNA) in neutrophils cultures. Neutrophils were stimulated by PMA (100 ng/mL) or Pyocyanin (10 µM), and DPBS as control for NET formation in various salt conditions. 150 000 neutrophils were seeded per well, and the fluorescence intensity of SYTOX™ Green Nucleic Acid stain was measured for 4 hours at 37°C and 5% CO_2_ in an Infinite F200 PRO plate reader (Tecan; ex. 485 nm, em. 535 nm). Endpoint values were analyzed.

To visualize NETs, we cultured neutrophils in chamber slides (Permanox, Thermo Fisher) for 4 hours at 37°C and 5% CO_2_ with PMA (100 ng/mL) or Pyocyanin (10 µM), or without stimuli (DPBS). We fixed the cells with 2% paraformaldehyde (PFA, Merck), permeabilized with 0.1% Triton X-100 (Merck), and blocked with blocking buffer (10% FCS, 2% BSA, 0.1% Triton X-100, 0.05% Tween 20 in DPBS) for 1 hour. Next, we stained them with primary rabbit anti-human neutrophil elastase (NE, Invitrogen, PA5-87158, 1:50), secondary goat anti-rabbit Cy5 (Jackson Lab., 111-175-144, 1:400) and DNA stain Hoechst33342 (Molecular Probes, 1µg/ml), and mounted them with DAKO fluorescence medium (Agilent). Controls were stained with Hoechst33342 and secondary goat anti-rabbit Cy5 only. We took the microphotographs with fluorescence microscope BZ-X710 (Keyence Corporation). Fluorescence microscopy pictures of NETs were analyzed using Adobe Photoshop CC 2018 to quantify the intensity of NE.

### 2.10 K/BxN-STA model for human inflammatory arthritis

We initially transferred the sera from arthritic K/BxN mice into healthy 2-month-old C57BL/6J female mice by intraperitoneal (i.p.) injection. The 3R principles (Replace, Reduce, Refine) were considered for the calculation of the minimum required sample size and it was determined that to have an 80% chance of detecting a drop of 4 points in the arthritis score at the 5% level of significance using 2-sided t-test, at least 5 mice per group were required to be included in this study. Simple (unrestricted) randomization of all mice into 4 groups followed. One group (n=8) was fed a diet with normal salt content (0.24% sodium) in food (ssniff Spezialdiäten GmbH, V1534-000) and water, while the other group (n=8) received a diet with high salt content: 4% NaCl-containing pellets (ssniff Spezialdiäten GmbH, E15431-34) and 0.9% NaCl-containing water one week before injection of the K/BxN serum and during the observation period. We followed the onset of arthritis for 43 days post-injection by measurement of ankle thickness with caliper and estimation of ankle thickness (clinical) by scoring of joint swelling of front and hind paws as follows: each paw was individually scored using a 4-point scale (0, normal paw; 1, swelling and redness of one joint type; 2, swelling and redness of two joint types; 3, swelling and redness on three joint types; 4, maximal swelling and redness which leads to complete joint deformity). Scoring was performed blindly and the measuring order of the cages was randomly alternated to avoid observer bias. Once the acute and resolution phases were defined, we induced arthritis to additional two groups (n=5) and euthanized at the peak of joint inflammation at day 10. The collected data were thereafter matched with the corresponding groups and analyzed accordingly.

### 2.11 Micro-computed tomography (µCT)

In order to evaluate bone erosions at distal sites from inflammation, we performed µCT imaging of tibiae collected after 43 days of STA was performed using the cone-beam Desktop Micro Computer Tomograph “µCT 40” by SCANCO Medical AG, Bruettisellen, Switzerland. The settings were optimized for calcified tissue visualization at 55 kVp with a current of 145 µA and 200 ms integration time for 500 projections/180°. For the segmentation of 3D-Volumes an isotropic voxel size of 8.4 µm and an evaluation script with adjusted greyscale thresholds of the operating system “Open VMS” by SCANCO Medical was used. For evaluation of the trabecular and bone structure of the proximal tibia metaphysis the volume of interest was determined as starting 0.42mm from the middle of the growth plate and extending 1.680mm (200 tomograms) distally. The segmentation of cortical and trabecular bone was carried out manually, based on a threshold dependent Open VMS-auto-contouring script provided by SCANCO Medical.

### 2.12 Histology

We euthanized 10 mice (5/group) on day 10 and fixed the paws overnight (ON) at RT in 4% PFA (Merck), washed them ON in 70% ethanol, and decalcified them in Teitel buffer for 2 weeks. Histological sections were prepared by embedding in paraffin and staining for hematoxylin and eosin (H&E) and tartrate-resistant acid phosphatase (TRAP). We took pictures with the fluorescence scanner (Aperio Versa 8, Leica Biosystems). We quantified the tissue area (T.Ar.), bone area (B.Ar.), inflammation area (Infl.Ar.), eroded area (Er.Ar.), and osteoclast count (N.Oc.) using the Aperio ImageScope image analysis software (Leica Biosystems). We stained another set of histological sections with primary antibody sheep anti-mouse neutrophil elastase (NE, R&D, AF4517, 1:100), secondary antibody donkey anti-sheep Alexa Fluor 647 (AF647, ThermoFisher Scientific, A-21448, 1:400) or primary antibody rat anti-mouse Ly6G (BioLegend, 127601, 1:100), secondary antibody donkey anti-rat Cy5 (712-175-150, Jackson Lab., 1:400) or primary antibody rabbit anti-human citrullinated histone H3 (citH3) (ab5103, Abcam, 1:300), secondary antibody goat anti-rabbit Cy5 (111-175-144, Jackson Lab., 1:400), and DAPI (1 µg/ml), followed by mounting with DAKO fluorescence medium. Controls were stained with DNA stain and secondary antibody only. We took microphotographs using the Aperio Versa 8 fluorescence scanner. We exported immunofluorescence images of the infiltrated areas of interest as grayscale TIFF files. We performed basic cell segmentation employing the Spectre R package (10.1002/cyto.a.24350), with instructions and source code provided at https://github.com/ImmuneDynamics/spectre. We used the stable version (4.2.1) of CellProfiler downloaded from the CellProfiler website (www.cellprofiler.org) to obtain single cell data. The fluorescence intensity of NE was further quantified using Adobe Photoshop CC 2018. Lastly, we performed NE activity measurement using deparaffinized paw sections from K/BxN mice as follows: we added the NE fluorogenic substrate (MeOSuc-AAPV-AMC, Santa Cruz Biotechnology, 1:10) to the tissue and incubated the slides at 37°C for 24 hours. The following day, we transferred the incubation fluid to a 96-well plate and measured the fluorescence intensity in an Infinite F200 PRO plate reader (Tecan; ex. 360 nm, em. 465 nm). We then took microphotographs employing the Aperio Versa 8 fluorescence scanner, and extracted the paw area using the Aperio ImageScope image analysis software (Leica Biosystems).

### 2.13 Murine osteoclast differentiation

We isolated macrophage-like osteoclast precursor cells from female C57BL/6J mice (6-14 weeks old) by flushing the bone marrow from femora and tibiae. We incubated the cells in a Petri dish at 37°C and 5% CO_2_ overnight. The next day, we collected the non-adherent cells and plated them on 96-well plates coated with calcium phosphate, at 1 x 106/mL (200 µL/well) in α-MEM (Gibco) containing 10% FCS and 1% Penicillin/Streptomycin, supplemented with 30 ng/mL recombinant murine M-CSF (Peprotech, 315-02) and 50 ng/mL recombinant murine sRANK Ligand (Peprotech, 315-11). Where stated, 5, 15, or 25 mM NaCl and/or 100 nM H_2_O_2_ were added in the medium change (every 2 days). On day 9, we fixed and stained the fully differentiated osteoclasts using the TRAP staining Kit (Merck, 387A-1KT). We identified purple colored cells with more than 3 nuclei as osteoclasts. One field per well was imaged. We quantified the osteoclasts employing the Carl Zeiss microscope equipped with a camera (Osteomeasure; Osteometrics). After TRAP analysis, cells were lysed with ddH2O, and we performed Von Kossa staining (Resorption Assay). We took the microphotographs of all wells using the fluorescence microscope BZ-X710. We identified the resorbed area as white patches on black background, and performed the morphometry analysis in ImageJ. We pooled the results from 5 mice.

### 2.14 Statistical analyses

We performed the statistical analyses in Excel 2019 (Microsoft) or in Graphpad Prism 9. We performed Mixed Effects Analysis, 2-way ANOVA, ordinary one-way ANOVA, Multiple t-tests, Unpaired t-test, Mann-Whitney test or Kruskal-Wallis test, as indicated in the figure legends. All data are presented as mean ± standard deviation (SD).

## 3 Results

**Figure 1:**
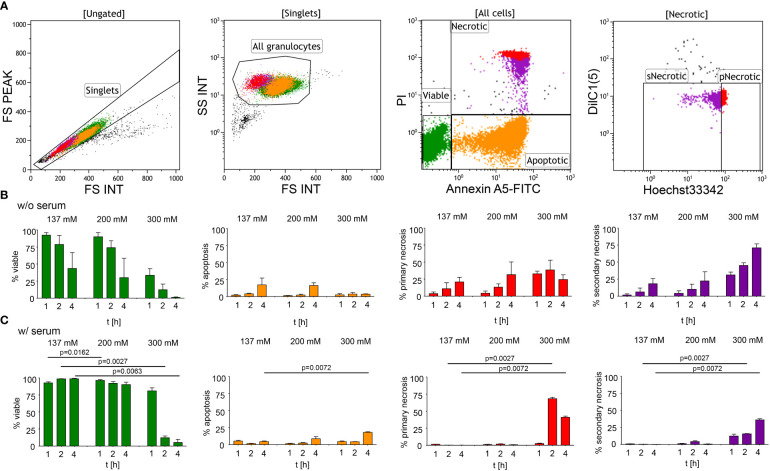
Hypertonic sodium-rich media decrease viability of human neutrophils and induce primary or secondary cell necrosis *in vitro*. Cell viability was assessed by flow cytometry after four-color death staining with PI, Hoechst33342, Annexin A5-FITC and DilC1(5) in various salt conditions (1) isotonicity 137 mM NaCl, (2) moderate hypertonicity 200 mM NaCl, and (3) high hypertonicity 300 mM NaCl, at various time points. **(A)** Flowcytometric dot plot analysis showing the gating strategy used to identify viable, apoptotic, and necrotic cell populations; **(B)** culture without (w/o) serum; **(C)** culture with (w/) 10% heat-inactivated FCS. Data were obtained from 3-5 healthy individuals and are presented as mean ± SD. Statistical analysis was performed using Mixed Effects Analysis. **Table 1** displays all numerical *P* values. pNecrotic, primary necrotic; sNecrotic, secondary necrotic.

To investigate the effect of hypertonic sodium-rich media on neutrophils, we first assessed the viability of human neutrophils *in vitro* in various tonicities by flow cytometry (**Figure 1A**): (1) isotonicity 137 mM NaCl, (2) moderate hypertonicity 200 mM NaCl, and (3) high hypertonicity 300 mM NaCl in either serum-free (**Figure 1B**) or serum-supplemented (**Figure 1C**) buffers. We induced hypertonicity by increasing the NaCl content by 63 mM (200 mM DPBS-NaCl), or 163 mM (300 mM DPBS-NaCl). Using a four-color death staining protocol (30) we differentiated the cell subpopulations by flow cytometry: viable (V), apoptotic (A), primary necrotic (pN), and secondary necrotic (sN) cells. High hypertonicity greatly reduced neutrophil viability, whereas moderate hypertonicity only marginally affected cell viability. In high hypertonicity, cell viability decreased greatly after 1 hour, and in serum-supplemented environment after 2 hours. Neutrophils underwent primary or secondary necrosis rather than apoptosis. **Table 1** depicts the differences in cell viability under various salt conditions.

**Table 1:** Cell viability^1^.

		P value (w/o serum)				P value (w/serum)		
	V	A	pN	sN	V	A	pN	sN
137 vs. 200 mM, 1h	n.s.	n.s.	n.s.	n.s.	0.0162	n.s.	n.s.	n.s.
137 vs. 300 mM, 1h	n.s.	n.s.	n.s.	n.s.	n.s.	n.s.	n.s.	n.s.
200 vs. 300 mM, 1h	n.s.	n.s.	n.s.	n.s.	n.s.	n.s.	n.s.	n.s.
137 vs. 200 mM, 2h	n.s.	n.s.	n.s.	n.s.	n.s.	n.s.	n.s.	n.s.
137 vs. 300 mM, 2h	n.s.	n.s.	n.s.	n.s.	0.0027	n.s.	0.0027	0.0027
200 vs. 300 mM, 2h	n.s.	n.s.	n.s.	n.s.	0.0036	n.s.	0.0027	0.0315
137 vs. 200 mM, 4h	n.s.	n.s.	n.s.	n.s.	n.s.	n.s.	n.s.	n.s.
137 vs. 300 mM, 4h	n.s.	n.s.	n.s.	n.s.	0.0063	0.0072	0.0072	0.0072
200 vs. 300 mM, 4h	n.s.	n.s.	n.s.	n.s.	0.0009	n.s.	0.0063	0.0054

^1^Statistical analysis was performed in Graphpad Prism 9 using Mixed Effects Analysis. All P values were calculated applying the Bonferroni correction; n.s., not significant.

**Figure 2:**
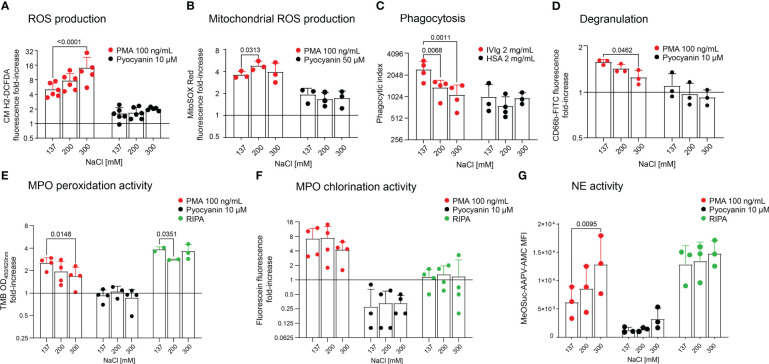
Neutrophils cultured in hypertonic sodium-rich media increase ROS production and NE activity, but reduce degranulation, phagocytosis and MPO peroxidation activity. Analyses of freshly isolated neutrophils in various salt conditions by flow cytometry: **(A)** ROS production; **(B)** Mitochondrial ROS production; **(C)** Phagocytosis; **(D)** Degranulation; **(E)** MPO peroxidation activity; **(F)** MPO chlorination activity, and **(G)** NE activity. In **(A, B, D–G)**, neutrophils were either not stimulated (DPBS) or stimulated with PMA (100 ng/mL) or Pyocyanin (10 µM or 50 µM). In **(C)**, cells were incubated with microspheres coated with either 2 mg/mL IVIg or 2 mg/mL HSA (baseline). In **(E)**, **(F)** and **(G)**, RIPA buffer was used for complete cell lysis and equals maximum enzymatic activity. All y-axes display log_2_ values, except in **(G)**. Data were obtained from 3-6 healthy individuals and are presented as mean ± SD. Statistical analysis was performed using 2-way ANOVA. OD, optical density; MFI, mean fluorescence intensity.

As cell death staining assays showed that neutrophils preserved viability in moderate hypertonicity, we tested whether this increase in salinity effects neutrophil effector functions. We measured ROS and mitochondrial ROS production upon stimulation with PMA or Pyocyanin (**Figures 2A, B**, respectively), and phagocytic activity in various salt conditions (**Figure 2C**). We further performed assays for degranulation (**Figure 2D**), MPO peroxidation and chlorination activity (**Figures 2E, F**, respectively) and observed that high hypertonicity (300 mM) increased ROS production upon stimulation (**Figure 2A**), and moderate hypertonicity (200 mM) increased mitochondrial ROS production. Likewise, in high hypertonicity (300 mM), NE activity was also increased (**Figure 2G**). On the contrary, phagocytosis (**Figure 2C**), degranulation (**Figure 2D**) and MPO peroxidation activity were significantly inhibited (**Figure 2E**) in high hypertonicity (300 mM). Importantly, phagocytosis was already affected by moderate hypertonicity (200 mM). The absolute values of unstimulated neutrophils are displayed in **Supplementary Figure S1**.

**Figure 3:**
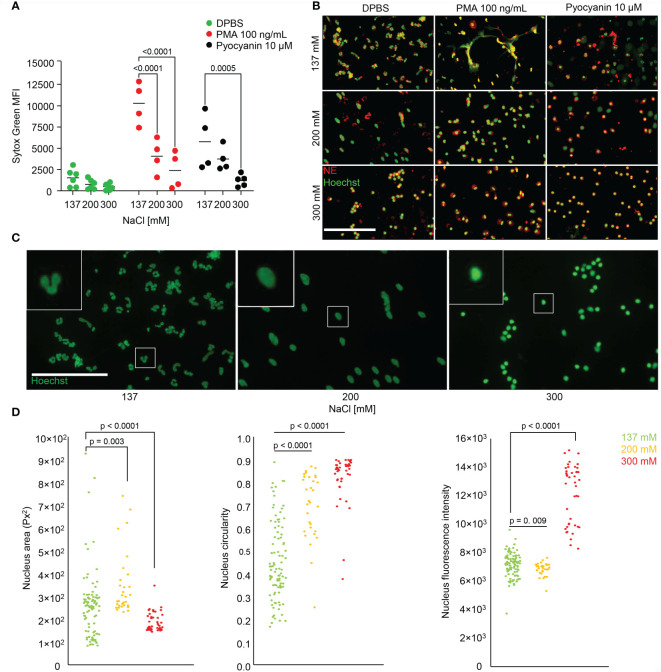
Hypertonic sodium-rich media inhibit NET formation *in vitro* and change neutrophils’ nuclear morphology. **(A, B)** NET formation in various salt conditions without serum unstimulated (DPBS), and after stimulation with PMA (100 ng/mL) or Pyocyanin (10 µM). Data were obtained from at least 4 healthy individuals and are presented as mean ± SD. Statistical analysis was performed using 2-way ANOVA; **(C, D)** nuclear morphology of unstimulated neutrophils stained with Hoechst33342 in various salt conditions and analysis by morphometry; statistical analysis was performed applying ordinary one-way ANOVA. MFI, mean fluorescence intensity; bars represent 100 µm.

We were next interested in the effect of salt on NET formation. We stimulated neutrophils with PMA or Pyocyanin to form NETs in various salt conditions (137, 200 or 300 mM), and used DPBS as a control (**Figures 3A, B**). We measured DNA externalization using the cell-impermeable SYTOX™ Green Nucleic Acid stain. NET formation was decreased with increasing salt concentrations. Upon PMA or Pyocyanin stimulation, NET formation was already reduced at 200 mM when compared to isotonic media. To confirm the suppression of NET formation, we employed fluorescence microscopy after immunostaining for NE in the presence of Hoechst33342 (**Figure 3B**). and analysed nuclear morphology and intracellular colocalization of DNA and NE. Unstimulated neutrophils in various salt conditions were also morphometrically evaluated (**Figure 3C**). To this end we compared area size, circularity and DNA content of the neutrophils’ nuclei (**Figure 3D**), and observed that the neutrophils lost their lobulated nuclear morphology (left panel) at moderate hypertonicity (middle panel). At high hypertonicity the nuclei shrunk and displayed increased Hoechst33342 fluorescence intensities (right panel).

**Figure 4:**
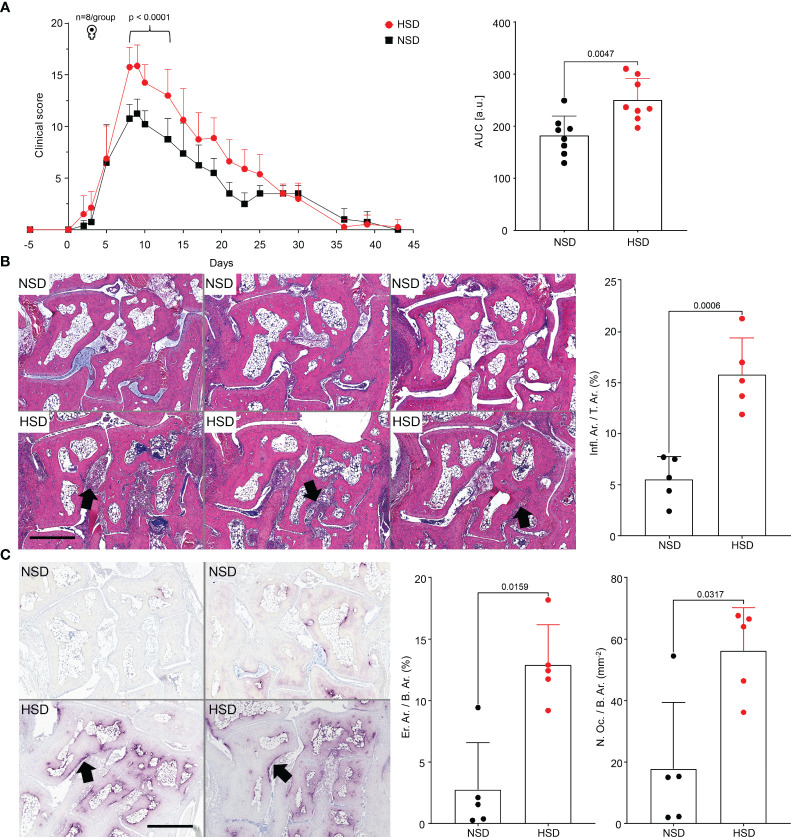
K/BxN mice kept on HSD develop more severe arthritis than mice on NSD. **(A)** Clinical scoring of front and hind paws in C57BL/6J mice with K/BxN serum transfer arthritis; corresponding plot of area under the curve (AUC). Data are shown from mice kept on either NSD, n=8, or HSD, n=8, and are presented as mean ± SD; **(B)** Representative images of HE-stained hind paw sections and their histological quantification at day 10. Upper row: paw sections of mice kept on NSD; lower row: paw sections of mice kept on HSD; **(C)** Representative images of TRAP-stained hind paw sections and their histological quantification. Upper row: left and right paw of a mouse kept on NSD; lower row: left and right paw of a mouse kept on HSD. Data are shown from mice kept on either NSD, n=5, or HSD, n=5, and are presented as mean ± SD. Statistical analysis was performed using 2-way ANOVA (Clinical Score), Unpaired t-test (Infl.Ar./T.Ar.) or Mann-Whitney test (AUC, Inflammation, Osteoclast Count). AUC, area under the curve; Infl.Ar., inflammation area; T.Ar., tissue area; Er.Ar., eroded area; B.Ar., bone area; N.Oc., osteoclast count; bars represent 900 µm. Black arrows indicate infiltrates **(B)**, or eroded bone **(C)**.

Next, we performed the murine STA model with a normal (NSD) or a high salt diet (HSD), to investigate the effect of salt on the development of arthritis *in vivo*. The course of arthritis was time-monitored (**Figure 4A**). Mice kept on HSD developed more severe arthritis, reflected by increased paw swelling and increased clinical score. The tibiae were scanned by µCT to investigate whether the inflammation of the paws caused changes in the proximal bone (**Figure S2**). Since µCT analysis revealed no long-term effect on tibial bone remodeling in HSD mice, we investigated the acute phase of arthritis by analyzing the hind paws with HE (**Figure 4B**) or TRAP staining (**Figure 4C**). Both techniques revealed significant morphological changes in mice on HSD with extended tissue inflammation (**Figure 4B**), increased bone erosion and osteoclast count (**Figure 4C**).

**Figure 5:**
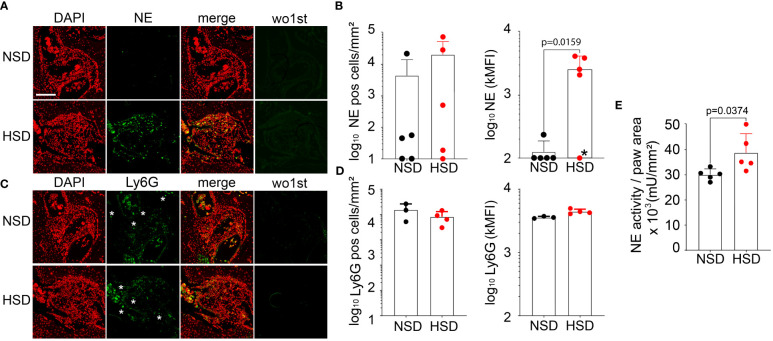
K/BxN mice on HSD are characterized by increased NE expression and enzymatic activity within the carpal bone infiltrates. Representative images of hind paw infiltrates **(A, C)** and their histological quantification **(B, D**, respectively**)**, after staining consecutive sections with primary antibody sheep anti-mouse NE (R&D, AF4517) or rat anti-mouse Ly6G (Biolegend, 127601) and DAPI. Corresponding controls (wo1st) were stained with secondary antibody only. Red: DAPI, green: NE or Ly6G, respectively. White asterisks indicate single cells. Pictures were taken using the fluorescence scanner (Aperio Versa 8, Leica Biosystems); **(E)** NE activity measurement in hind paw sections. Data are presented as mean ± SD. Statistical analysis was performed using Mann-Whitney test (NE MFI) or Unpaired t-test (NE activity); outlier marked with asterisk was excluded. MFI, mean fluorescence intensity; pos, positive; bar represents 200 µm.

We analyzed hind paw sections for NE, Ly6G (**Figure 5**) and citrullinated histone H3 (citH3) by fluorescence microscopy (**Figure S3**). Between the carpal bones we detected extracellular DNA, co-located with citH3 or NE, namely NETs (**Figure S3A–D**, respectively). Importantly, we also detected intact neutrophils (Ly6G positive cells) infiltrating the carpal bones of both mice kept on NSD or HSD (**Figures 5C, D**). Infiltrating neutrophils displayed a granular phenotype with no obvious signs of NET formation. However, quantification of similar sized bone infiltrates from mice on NSD or HSD revealed that the frequency of NE positive cells tended to be higher in HSD mice, and that the intensity of NE expression at the single cell level was increased in the HSD group (**Figures 5A, B**). Accordingly, the NE activity was increased in the paws of HSD mice (**Figure 5E**).

**Figure 6:**
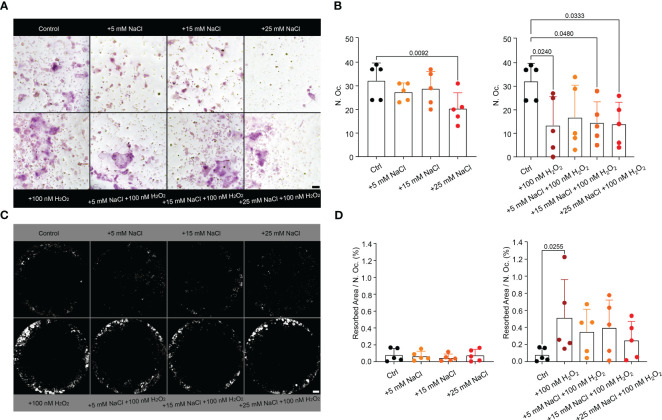
Hypertonic NaCl, as well as external ROS (H_2_O_2_) impair the maturation of murine osteoclasts *in vitro*. H_2_O_2_ increases osteoclast resorption activity *in vitro*. **(A)** TRAP-stained osteoclasts (n=5 per group) in purple (>3 nuclei) and **(B)** their quantification. Statistical analysis was performed using Kruskal-Wallis test; N. Oc., osteoclast count; bar represents 50 µM; **(C)** Von Kossa staining of the corresponding area resorbed by the osteoclasts (white) and **(D)** quantification per osteoclast (%), n=5. Data are presented as mean ± SD. Statistical analysis was performed using Kruskal-Wallis test. N.Oc., osteoclast count; bars represent 500 µM.

Since neutrophil infiltrations in a high salt environment were accompanied by more severe arthritis, increased osteoclast counts and bone erosion, and neutrophils responded to high salt with increased ROS production, we analyzed the effect of NaCl, H_2_O_2_ (ROS mimetic) or both on osteoclast differentiation (**Figures 6A, B**) and their resorptive activity (**Figures 6C, D**) *in vitro*. The addition of 25 mM NaCl or 100 nM H_2_O_2_ decreased total osteoclast counts when compared to controls. Whereas NaCl alone had no effect, the addition of H_2_O_2_ increased osteoclast resorption.

## 4 Discussion

Diet together with other environmental factors accounts for 70% of the risk towards developing an autoimmune disease (31). HSD is considered to be generally pro-inflammatory, but more recent findings imply that it has anti-inflammatory effects as well, such as polarizing the medullary mononuclear phagocytes, and inducing neutrophil apoptosis (32, 33). High salt conditions not only cause osmotic and ionic imbalance, but also oxidative stress and metabolic changes (34). In our work, we assessed the effect of hypertonic sodium-rich media on neutrophil viability and activity in various salt conditions *in vitro*. Our data show that the high salt content compromised neutrophil-driven immune response by decreasing neutrophil viability, degranulation, phagocytosis, MPO activity and NET formation; processes crucial for immune defense (35). However, high salt content increased oxidative burst activity, an effective but damaging mechanism of defense. Moderate salt content increased mitochondrial ROS production. Nuclear morphometry of unstimulated neutrophils revealed that at 200 mM NaCl the nuclei rounded up and swelled. At 300 mM NaCl nuclei shrunk, got more circular, and displayed a higher fluorescence intensity, indicating higher DNA densities in the pyknotic nuclei. Pyknosis is known as the irreversible condensation of chromatin in the nucleus of a cell undergoing cell death and precedes karyorrhexis (36, 37). Our data indicate that neutrophils in hypertonic environment initiated the apoptotic program with preserved membrane integrity, rapid loss of mitochondrial potential, and shrinkage of the nucleus before they progressed to secondary necrosis. Interestingly, under these conditions the release of DNA was abrogated.

In the work of Nadesalingam et al., hypertonic saline induced neutrophil apoptosis along with decrease in ROS production and NET formation (17). Our data rather suggest that while the hypertonic environment promoted apoptotic cell death and suppressed NET formation, the ability to produce ROS was preserved, or enhanced. An enhanced ROS production due to metabolic imbalances is used by cells to sense stress and was also reported by other authors due to osmotic stress and increased salinity (34, 38). Superoxide production was lower when neutrophils were pre-treated with hypertonic saline and then activated, and highest, when cells were first activated, and then treated with hypertonic saline. Meaning, the increased ROS production driven by high salt depends on prior activation of the neutrophils. Neutrophil cytotoxicity is therefore increased by higher salinity only if the system has already been primed by inflammation and cells were pre-activated (39). In our settings, neutrophils were simultaneously activated by PMA/Pyocyanin and treated with high salt; these conditions augmented ROS production in high hypertonicity. This is in line with recent findings where NaCl initially inhibits (up to 2 hours) (18, 40) but later (6 to 18 hours) fosters ROS production (18), even at lower osmolarities (190 - 200 mM). Our higher salinity was enough to induce oxidative stress in short-term cell cultures. Whereas high hypertonicity (300 mM) increased the oxidative burst, already moderate hypertonicity (200 mM) increased the ROS production in the mitochondria of neutrophils. Interestingly, NET formation, in which also mitochondrial ROS can be used as an alternative ROS source in the absence of functional NADPH-oxidase (41), was decreased. Both NOX-dependent and NOX- independent NET formation (42) were abrogated despite increased levels of ROS, and mitochondrial ROS, respectively. Mitochondrial ROS also plays an important role in cells´ signalling pathways, including the regulation of immune responses, apoptosis, autophagy and inflammation (43). Once the cellular homeostasis is disrupted, as with increased NaCl content in our settings, mitochondrial ROS facilitate crosstalk to determine the cell´s fate. Taken together, neutrophils display a higher oxidative stress level and a rapid progression to secondary necrosis in high hypertonicity.

In RA, several immune-mediated mechanisms affect the balance of bone-forming osteoblasts and bone-resorbing osteoclasts (44). Excessive osteoclast activity has been linked to bone erosion, joint destruction and disability. Recent findings revealed that for osteoclast differentiation and resorption activity, ROS are necessary. The application of oxidant scavengers, such as N-acetylcysteine (NAC) or diphenylene iodonium (DPI) suppressed RANKL-mediated ROS production, and consequently inhibited osteoclast differentiation (45). Importantly, RANKL KO mice are protected from bone erosion after the transfer of K/BxN serum (46). As our *in vitro* data showed an increased ROS production by neutrophils in high hypertonicity, we examined the effect of HSD in the STA arthritis model. In line with previous findings, our data show that HSD boosted osteoclast differentiation and bone erosion as seen in bone histology of osteoclast-specific TRAP staining (47). The aggravated arthritis in the STA mice on HSD was accompanied by high osteoclast count (N.Oc./B.Ar.), increased bone erosion (Er.Ar./B.Ar.), and increased inflamed tissue area (Infl.Ar./T.Ar.).

In this work, neutrophils infiltrated the interosseous spaces of the STA mice, especially in HSD. This likely contributed to the release of pro-inflammatory mediators leading to inflammation and arthritis aggravation. Whether this was a direct effect of NaCl, or an indirect effect mediated by increased ROS production of neutrophils was examined in a simplified *in vitro* system. As in the *in vitro* studies using human neutrophils, various NaCl conditions were tested in experiments using *in vitro* generated osteoclasts. Our findings indicated that H_2_O_2_ alone, increased osteoclasts’ resorptive activity. Therefore, we suggest that the accumulation of neutrophils in inflamed synovium is an important source of ROS promoting osteoclast differentiation (48).

This study examined the relation between NaCl, neutrophils and osteoclasts both *in vitro* and *in vivo*. It investigated the effect of increased hypertonicity on neutrophils in more detail, and how this affects osteoclast differentiation and function in the context of RA. Our study has some limitations, and to clarify the exact relations between neutrophils and osteoclasts, further experiments need to be conducted. These include neutrophil depletion *in vivo* in a STA model with mice on NSD/HSD and the use of ROS scavengers such as NAC. Based on our data, we postulate that the increased osteoclast differentiation and bone erosion observed in arthritis under HSD are not a direct effect of NaCl on osteoclast differentiation and activity, but rather a consequence of the increased local ROS production by the infiltrating neutrophils in a hypertonic environment. HSD contributed to an enhanced oxidative milieu maintained by infiltrating neutrophils, which greatly aggravated arthritis by supporting bone erosion.

## Author’s note

This manuscript will be used as part of Leticija Zlatar´s doctoral thesis.

## Data availability statement

The original contributions presented in the study are included in the article/**Supplementary Material**. Further inquiries can be directed to the corresponding author.

## Ethics statement

The studies involving human participants were reviewed and approved by Ethical committee of the University Hospital Erlangen (permit 243_15 B). The patients/participants provided their written informed consent to participate in this study. The animal study was reviewed and approved by Local Animal Care and Use Committees of the Danylo Halytsky Lviv National Medical University (permit numbers 20191219/10 and 20201221/9).

## Author contributions

MH, RB and JT conceived the project. JK and LM designed and supervised the study. US, MHH and GS supported the work with their expertise. LZ, AM, GB and DW performed the experiments. LZ, AM, and MM-B analyzed the data. LZ wrote the original manuscript. JK, LM and MH revised it. All authors have read and agreed to the published version of the manuscript. All authors contributed to the article and approved the submitted version.

## References

[1] Hahn J Knopf J Maueroder C Kienhofer D Leppkes M Herrmann M. Neutrophils and neutrophil extracellular traps orchestrate initiation and resolution of inflammation. Clin Exp Rheumatol (2016) 34(4 Suppl 98):6–8. 27586795

[2] Delgado-Rizo V Martinez-Guzman MA Iniguez-Gutierrez L Garcia-Orozco A Alvarado-Navarro A Fafutis-Morris M. Neutrophil extracellular traps and its implications in inflammation: an overview. Front Immunol (2017) 8:81. doi: 10.3389/fimmu.2017.00081 28220120 PMC5292617

[3] Grigorieva DV Gorudko IV Sokolov AV Kostevich VA Vasilyev VB Cherenkevich SN. Myeloperoxidase stimulates neutrophil degranulation. Bull Exp Biol Med (2016) 161(4):495–500. doi: 10.1007/s10517-016-3446-7 27597056

[4] Strzepa A Pritchard KA Dittel BN. Myeloperoxidase: A new player in autoimmunity. Cell Immunol (2017) 317:1–8. doi: 10.1016/j.cellimm.2017.05.002 28511921 PMC5665680

[5] Schauer C Janko C Munoz LE Zhao Y Kienhofer D Frey B. Aggregated neutrophil extracellular traps limit inflammation by degrading cytokines and chemokines. Nat Med (2014) 20(5):511–7. doi: 10.1038/nm.3547 24784231

[6] Estua-Acosta GA Zamora-Ortiz R Buentello-Volante B Garcia-Mejia M Garfias Y. Neutrophil extracellular traps: current perspectives in the eye. Cells (2019) 8(9). doi: 10.3390/cells8090979 PMC6769795 31461831

[7] Song W Ye J Pan N Tan C Herrmann M. Neutrophil extracellular traps tied to rheumatoid arthritis: points to ponder. Front Immunol (2020) 11:578129. doi: 10.3389/fimmu.2020.578129 33584645 PMC7878527

[8] Wright HL Moots RJ Bucknall RC Edwards SW. Neutrophil function in inflammation and inflammatory diseases. Rheumatol (Oxford) (2010) 49(9):1618–31. doi: 10.1093/rheumatology/keq045 20338884

[9] Monach PA Mathis D Benoist C. The K/BxN arthritis model. Curr Protoc Immunol (2008) 15(22): 1–15. doi: 10.1002/0471142735.im1522s81 18491295

[10] Wipke BT Allen PM. Essential role of neutrophils in the initiation and progression of a murine model of rheumatoid arthritis. J Immunol (2001) 167(3):1601–8. doi: 10.4049/jimmunol.167.3.1601 11466382

[11] Kleinewietfeld M Manzel A Titze J Kvakan H Yosef N Linker RA. Sodium chloride drives autoimmune disease by the induction of pathogenic TH17 cells. Nature (2013) 496(7446):518–22. doi: 10.1038/nature11868 PMC3746493 23467095

[12] Wilck N Matus MG Kearney SM Olesen SW Forslund K Bartolomaeus H. Salt-responsive gut commensal modulates T(H)17 axis and disease. Nature (2017) 551(7682):585–9. doi: 10.1038/nature24628 PMC6070150 29143823

[13] Scrivo R Perricone C Altobelli A Castellani C Tinti L Conti F. Dietary habits bursting into the complex pathogenesis of autoimmune diseases: the emerging role of salt from experimental and clinical studies. Nutrients (2019) 11(5). doi: 10.3390/nu11051013 PMC6566149 31060286

[14] Toussirot E Bereau M Vauchy C Saas P. Could sodium chloride be an environmental trigger for immune-mediated diseases? An overview of the experimental and clinical evidence. Front Physiol (2018) 9:440. doi: 10.3389/fphys.2018.00440 29740348 PMC5928237

[15] Jung SM Kim Y Kim J Jung H Yi H Rim YA. Sodium chloride aggravates arthritis via Th17 polarization. Yonsei Med J (2019) 60(1):88–97. doi: 10.3349/ymj.2019.60.1.88 30554495 PMC6298894

[16] Matsumoto T van der Auwera P Watanabe Y Tanaka M Ogata N Naito S. Neutrophil function in hyperosmotic NaCl is preserved by phosphoenol pyruvate. Urol Res (1991) 19(4):223–7. doi: 10.1007/BF00305299 1656579

[17] Nadesalingam A Chen JHK Farahvash A Khan MA. Hypertonic saline suppresses NADPH oxidase-dependent neutrophil extracellular trap formation and promotes apoptosis. Front Immunol (2018) 9:359. doi: 10.3389/fimmu.2018.00359 29593709 PMC5859219

[18] Mazzitelli I Bleichmar L Melucci C Gerber PP Toscanini A Cuestas ML. High salt induces a delayed activation of human neutrophils. Front Immunol (2022) 13:831844. doi: 10.3389/fimmu.2022.831844 35720394 PMC9204211

[19] Danziger J Zeidel ML. Osmotic homeostasis. Clin J Am Soc Nephrol (2015) 10(5):852–62. doi: 10.2215/CJN.10741013 PMC4422250 25078421

[20] Sharif K Amital H Shoenfeld Y. The role of dietary sodium in autoimmune diseases: The salty truth. Autoimmun Rev (2018) 17(11):1069–73. doi: 10.1016/j.autrev.2018.05.007 30213699

[21] Whelton PK He J. Health effects of sodium and potassium in humans. Curr Opin Lipidol (2014) 25(1):75–9. doi: 10.1097/MOL.0000000000000033 24345983

[22] Sadowski J Dobrowolski L. The renal medullary interstitium: focus on osmotic hypertonicity. Clin Exp Pharmacol Physiol (2003) 30(3):119–26. doi: 10.1046/j.1440-1681.2003.03810.x 12603338

[23] Jantsch J Schatz V Friedrich D Schroder A Kopp C Siegert I. Cutaneous Na+ storage strengthens the antimicrobial barrier function of the skin and boosts macrophage-driven host defense. Cell Metab (2015) 21(3):493–501. doi: 10.1016/j.cmet.2015.02.003 25738463 PMC4350016

[24] Borthakur A Shapiro EM Akella SV Gougoutas A Kneeland JB Reddy R. Quantifying sodium in the human wrist in *vivo* by using MR imaging. Radiology (2002) 224(2):598–602. doi: 10.1148/radiol.2242011039 12147862

[25] Ouwerkerk R Bleich KB Gillen JS Pomper MG Bottomley PA. Tissue sodium concentration in human brain tumors as measured with 23Na MR imaging. Radiology (2003) 227(2):529–37. doi: 10.1148/radiol.2272020483 12663825

[26] Salgado E Bes-Rastrollo M de Irala J Carmona L Gomez-Reino JJ. High sodium intake is associated with self-reported rheumatoid arthritis: a cross sectional and case control analysis within the SUN cohort. Med (Baltimore) (2015) 94(37):e0924. doi: 10.1097/MD.0000000000000924 PMC4635786 26376372

[27] Marouen S du Cailar G Audo R Lukas C Vial G Tournadre A. Sodium excretion is higher in patients with rheumatoid arthritis than in matched controls. PloS One (2017) 12(10):e0186157. doi: 10.1371/journal.pone.0186157 29028829 PMC5640209

[28] Dmitrieva NI Burg MB. Hypertonic stress response. Mutat Res (2005) 569(1-2):65–74. doi: 10.1016/j.mrfmmm.2004.06.053 15603752

[29] Go WY Liu X Roti MA Liu F Ho SN. NFAT5/TonEBP mutant mice define osmotic stress as a critical feature of the lymphoid microenvironment. Proc Natl Acad Sci U S A (2004) 101(29):10673–8. doi: 10.1073/pnas.0403139101 PMC489993 15247420

[30] Munoz LE Maueroder C Chaurio R Berens C Herrmann M Janko C. Colourful death: six-parameter classification of cell death by flow cytometry–dead cells tell tales. Autoimmunity (2013) 46(5):336–41. doi: 10.3109/08916934.2012.755960 23231469

[31] Azizov V Dietel K Steffen F Durholz K Meidenbauer J Lucas S. Ethanol consumption inhibits TFH cell responses and the development of autoimmune arthritis. Nat Commun (2020) 11(1):1998. doi: 10.1038/s41467-020-15855-z 32332730 PMC7181688

[32] Jobin K Stumpf NE Schwab S Eichler M Neubert P Rauh M. A high-salt diet compromises antibacterial neutrophil responses through hormonal perturbation. Sci Transl Med (2020) 12(536). doi: 10.1126/scitranslmed.aay3850 32213629

[33] Kim JY Hong YS Choi SH Yoon YH Moon SW Lee SW. Effect of hypertonic saline on apoptosis of polymorphonuclear cells. J Surg Res (2012) 178(1):401–8. doi: 10.1016/j.jss.2012.01.055 22475352

[34] Hossain MS Dietz KJ. Tuning of redox regulatory mechanisms, reactive oxygen species and redox homeostasis under salinity stress. Front Plant Sci (2016) 7:548. doi: 10.3389/fpls.2016.00548 27242807 PMC4861717

[35] Hampton MB Chambers ST Vissers MC Winterbourn CC. Bacterial killing by neutrophils in hypertonic environments. J Infect Dis (1994) 169(4):839–46. doi: 10.1093/infdis/169.4.839 8133099

[36] Hou L Liu K Li Y Ma S Ji X Liu L. Necrotic pyknosis is a morphologically and biochemically distinct event from apoptotic pyknosis. J Cell Sci (2016) 129(16):3084–90. doi: 10.1242/jcs.184374 27358477

[37] Burgoyne LA. The mechanisms of pyknosis: hypercondensation and death. Exp Cell Res (1999) 248(1):214–22. doi: 10.1006/excr.1999.4406 10094828

[38] Miller G Suzuki N Ciftci-Yilmaz S Mittler R. Reactive oxygen species homeostasis and signalling during drought and salinity stresses. Plant Cell Environ (2010) 33(4):453–67. doi: 10.1111/j.1365-3040.2009.02041.x 19712065

[39] Ciesla DJ Moore EE Zallen G Biffl WL Silliman CC. Hypertonic saline attenuation of polymorphonuclear neutrophil cytotoxicity: timing is everything. J Trauma (2000) 48(3):388–95. doi: 10.1097/00005373-200003000-00004 10744274

[40] Krampert L Bauer K Ebner S Neubert P Ossner T Weigert A. High Na(+) environments impair phagocyte oxidase-dependent antibacterial activity of neutrophils. Front Immunol (2021) 12:712948. doi: 10.3389/fimmu.2021.712948 34566968 PMC8461097

[41] Lood C Blanco LP Purmalek MM Carmona-Rivera C De Ravin SS Smith CK. Neutrophil extracellular traps enriched in oxidized mitochondrial DNA are interferogenic and contribute to lupus-like disease. Nat Med (2016) 22(2):146–53. doi: 10.1038/nm.4027 PMC4742415 26779811

[42] Douda DN Khan MA Grasemann H Palaniyar N. SK3 channel and mitochondrial ROS mediate NADPH oxidase-independent NETosis induced by calcium influx. Proc Natl Acad Sci U S A (2015) 112(9):2817–22. doi: 10.1073/pnas.1414055112 PMC4352781 25730848

[43] Dan Dunn J Alvarez LA Zhang X Soldati T. Reactive oxygen species and mitochondria: A nexus of cellular homeostasis. Redox Biol (2015) 6:472–85. doi: 10.1016/j.redox.2015.09.005 PMC4596921 26432659

[44] Harre U Lang SC Pfeifle R Rombouts Y Fruhbeisser S Amara K. Glycosylation of immunoglobulin G determines osteoclast differentiation and bone loss. Nat Commun (2015) 6:6651. doi: 10.1038/ncomms7651 25825024 PMC4389255

[45] Chen K Qiu P Yuan Y Zheng L He J Wang C. Pseurotin A inhibits osteoclastogenesis and prevents ovariectomized-induced bone loss by suppressing reactive oxygen species. Theranostics (2019) 9(6):1634–50. doi: 10.7150/thno.30206 PMC6485188 31037128

[46] Pettit AR Ji H von Stechow D Muller R Goldring SR Choi Y. TRANCE/RANKL knockout mice are protected from bone erosion in a serum transfer model of arthritis. Am J Pathol (2001) 159(5):1689–99. doi: 10.1016/S0002-9440(10)63016-7 PMC1867076 11696430

[47] Schroder A Gubernator J Leikam A Nazet U Cieplik F Jantsch J. Dietary salt accelerates orthodontic tooth movement by increased osteoclast activity. Int J Mol Sci (2021) 22(2). doi: 10.3390/ijms22020596 PMC7827744 33435280

[48] Agidigbi TS Kim C. Reactive oxygen species in osteoclast differentiation and possible pharmaceutical targets of ROS-mediated osteoclast diseases. Int J Mol Sci (2019) 20(14). doi: 10.3390/ijms20143576 PMC6678498 31336616

