# Supplementary material for: Suppression of neutrophils by sodium exacerbates oxidative stress and arthritis
Source: Front Immunol. 2023 Aug 2;14:1174537. doi: 10.3389/fimmu.2023.1174537 (PMC10433750; doi:10.3389/fimmu.2023.1174537)
Supplement: Supplementary file 1 [file DataSheet_1.docx]

Supplementary Material

Suppression of neutrophils by sodium exacerbates oxidative stress and arthritis

Leticija Zlatar, Aparna Mahajan, Marco Munoz-Becerra, Daniela Weidner, Galyna Bila, Rostyslav Bilyy, Jens Titze, Markus Hoffmann, Georg Schett, Martin Herrmann, Ulrike Steffen, Luis E. Munoz^*^, and Jasmin Knopf

*** Correspondence:** Luis E. Munoz: Luis.Munoz@fau.de

**
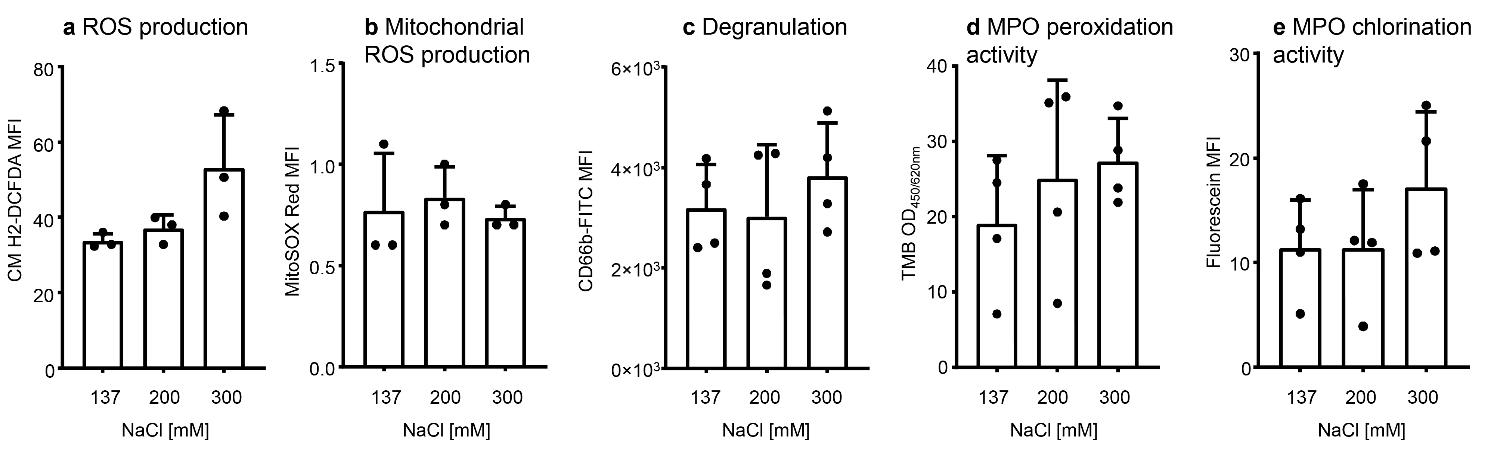
Supplementary Figure 1. Absolute values of various effector functions of unstimulated neutrophils.** a ROS production; b Mitochondrial ROS production; c Degranulation; d MPO peroxidation activity; e MPO chlorination activity from freshly isolated, unstimulated neutrophils in various salt conditions (1) isotonicity 137 mM NaCl, (2) moderate hypertonicity 200 mM NaCl, and (3) high hypertonicity 300 mM NaCl. Data were obtained from 3-4 healthy individuals. Statistical analysis was performed using Ordinary one-way ANOVA; abbreviations: MFI: mean fluorescence intensity, OD: optical density.


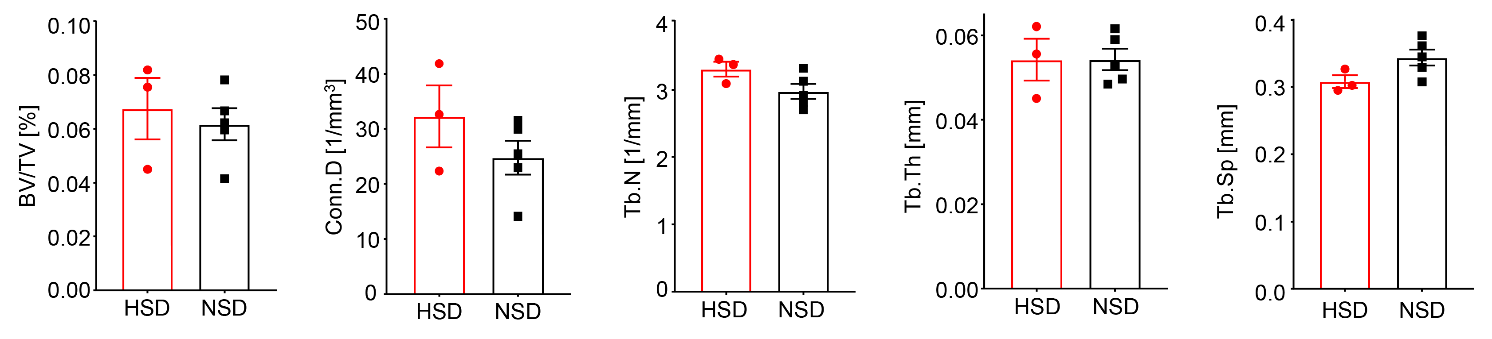
**Supplementary Figure 2. HSD has no long-term effect on tibial bone remodeling.** µCT analysis of tibiae from mice euthanized on day 43. Abbreviations: BV/TV: bone volume fraction, Conn.D: connectivity density; Tb.N: trabecular number; Tb.Th: trabecular thickness; Tb.Sp: trabecular separation.


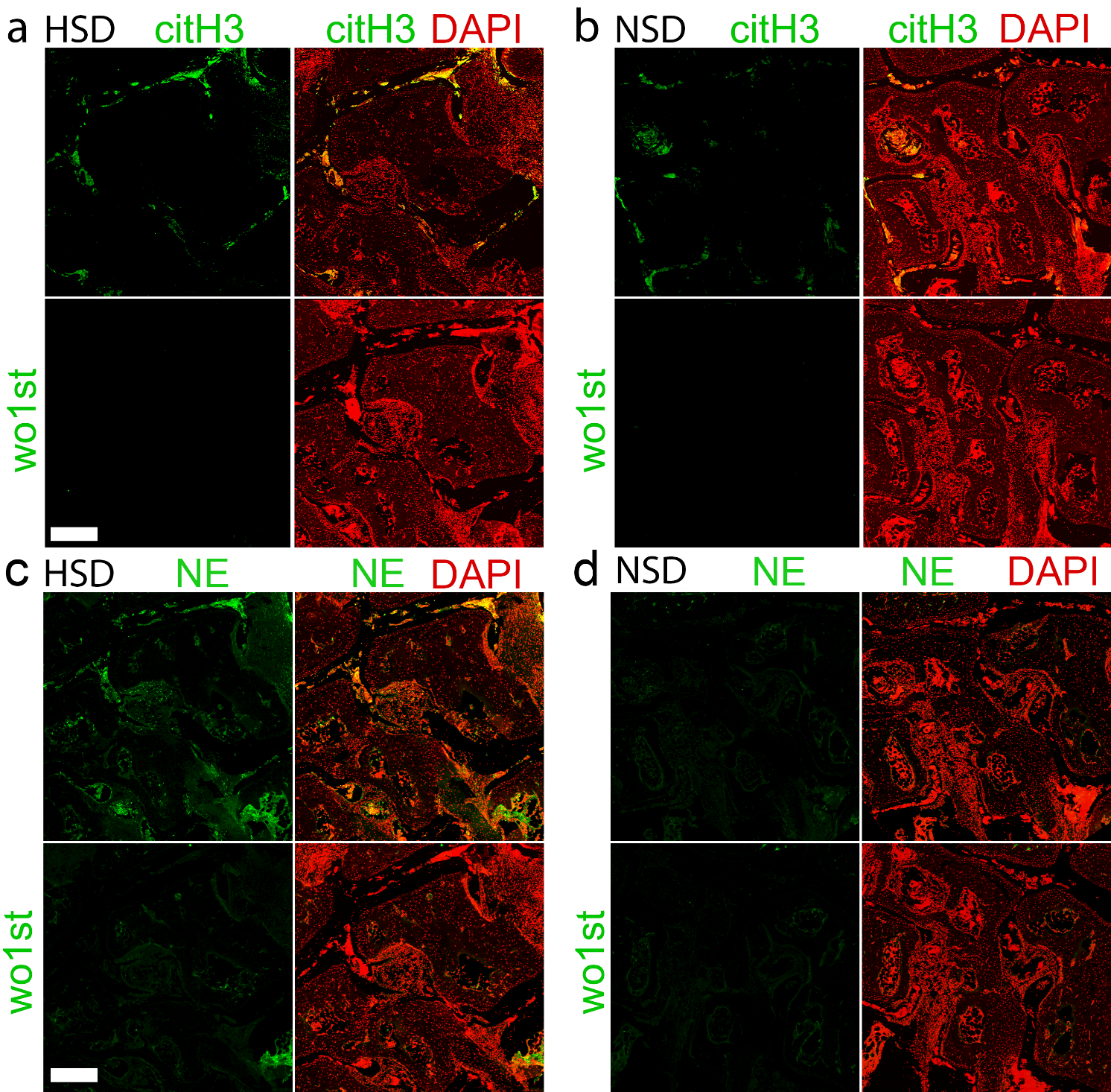


**Supplementary Figure 3.** **NETs infiltrate interosseous spaces of K/BxN mice.** Representative images of hind paws: a,c HSD; b,d NSD, after staining with primary antibody rabbit anti-human citH3 (ab5103, Abcam) or NE (R&D, AF4517) and DAPI. Corresponding controls were stained with secondary antibody only. Red: DAPI, green: citrullinated histone H3 or NE, respectively. Pictures were taken using the fluorescence scanner (Aperio Versa 8, Leica Biosystems). Statistical analysis was performed using Mann-Whitney test; bar represents 500 µm.
